# Response from the authors to the letter to the editor ‒ Pulmonary arterial catheter vs. prediction index software in patients undergoing orthotopic liver transplantation: “We cannot lump together everything”

**DOI:** 10.1016/j.bjane.2025.844620

**Published:** 2025-04-03

**Authors:** Jacek B. Cywinski, co-authors of the study

**Affiliations:** a Cleveland Clinic, Department of General Anesthesiology, Cleveland, Ohio; b Cleveland Clinic, Department of Outcome Research, Cleveland, Ohio

Dear Editor,

We appreciate Vetrugno's thoughtful comments regarding our article, “Evaluation of Hypotension Prediction Index Software in Patients Undergoing Orthotopic Liver Transplantation: A Retrospective Observational Study”.^1^ The points raised are valid; however, they should be considered in the context of our study's stated objectives and limitations.

Our investigation aimed to assess the agreement between the Pulmonary Artery Catheter (PAC) and the Hypotension Prediction Index (HPI) software in patients undergoing Liver Transplantation (LT), specifically estimation of Cardiac Output (CO) and Systemic Vascular Resistance (SVR).

We did not attempt to make a practice recommendations as to which LT patients benefit from PAC and in what clinical scenarios PAC can be substituted by different modality to measure CO. Use of PAC varies across transplant centers, and some clinicians may use a high MELD score as an indication for its placement, others may question this indication, for instance in our program, PAC is used in all cases alongside Transesophageal Echocardiography (TEE) and arterial waveform contour analysis. This study was not designed to validate or discredit any specific indications for PAC, nor to prescribe monitoring strategies for LT patients. Instead, it focused on comparing a gold standard (PAC) with a relatively new technology (HPI) on agreement of measured CO and SVR.

We fully agree with Vetrugno that LT patients present with a complex hemodynamic profile, involving vascular tone abnormalities, microvascular dysfunction, cirrhotic cardiomyopathy, post reperfusion syndrome, and abrupt preload changes during Inferior Vena Cava (IVC) clamping. Given these challenges, we sought to evaluate the agreement between PAC derived thermodilution CO, a valid method to measure CO in patients with hyperdynamic circulation, and HPI software, which, to our knowledge, has not been previously tested in LT patient. Since LT patients typically exhibit normal or supranormal CO, we selected an arbitrary but clinically practical precision threshold of ±20% for agreement between the two methods. We left up to the reader to decide if the degree of disagreement, would disqualify or prove to be acceptable in patients with hyperdynamic circulation. Certainly, accepting more than 40% disagreement between PAC (gold standard) and HPI software as acceptable, poses a risk of misguided therapy, at least in our opinion.

The prognostic accuracy of HPI has been validated in previous studies and was not the primary focus of our investigation. Instead, we aimed to build on our prior findings by using an HPI threshold of ≥ 85 to define alert episodes and assessing its effectiveness in predicting subsequent hypotension in LT patients.^2^ While evaluating HPI accuracy across different LT phases is an intriguing suggestion, our sample size was too small to draw robust conclusions. Furthermore, hypotension during LT arises from various mechanisms ‒ including hypovolemia, vasodilation, and impaired cardiac contractility ‒ so for HPI to be clinically useful, it must predict hypotension and identify its cause across all LT phases. A device accurate only in specific LT phases would have limited practical value and high rate of false positive alerts may be quite disruptive, limiting application of this technology. Similarly, our study was underpowered to assess HPI accuracy across specific MELD score ranges. Conceptually, for the HPI software to be helpful during LT, it should provide reliable prediction across entire range of the MELD scores and all phases of the surgery. This is another reason why assessing HPI in subsets of the patients undergoing LT may not be practical.

We appreciate the opportunity to clarify these points and welcome further discussion on this important topic.

## Authors' contributions

Jacek B. Cywinski: This author prepared the response to the Letter to the Editor.

## Conflicts of interest

The author declares no conflicts of interest.
